# Enhanced SQL injection detection using chi-square feature selection and machine learning classifiers

**DOI:** 10.3389/fdata.2025.1686479

**Published:** 2025-11-19

**Authors:** Emanuel Casmiry, Neema Mduma, Ramadhani Sinde

**Affiliations:** Computational and Communication Science and Engineering (CoCSE), The Nelson Mandela African Institution of Science and Technology (NM-AIST), Arusha, Tanzania

**Keywords:** cyberattacks, machine learning, feature selection, SQL injection, high-dimensional data

## Abstract

In the face of increasing cyberattacks, Structured Query Language (SQL) injection remains one of the most common and damaging types of web threats, accounting for over 20% of global cyberattack costs. However, due to its dynamic and variable nature, the current detection methods often suffer from high false positive rates and lower accuracy. This study proposes an enhanced SQL injection detection using Chi-square feature selection (FS) and machine learning models. A combined dataset was assembled by merging a custom dataset with the SQLiV3.csv file from the Kaggle repository. A Jensen–Shannon Divergence (JSD) analysis revealed moderate domain variation (overall JSD = 0.5775), with class-wise divergence of 0.1340 for SQLi and 0.5320 for benign queries. Term Frequency-Inverse Document Frequency (TF-IDF) was used to convert SQL queries into feature vectors, followed by the Chi-square feature selection to retain the most statistically significant features. Five classifiers, namely multinomial Naïve Bayes, support vector machine, logistic regression, decision tree, and K-nearest neighbor, were tested before and after feature selection. The results reveal that Chi-square feature selection improves classification performance across all models by reducing noise and eliminating redundant features. Notably, Decision Tree and K-Nearest Neighbors (KNN) models, which initially performed poorly, showed substantial improvements after feature selection. The Decision Tree improved from being the second-worst performer before feature selection to the best classifier afterward, achieving the highest accuracy of 99.73%, precision of 99.72%, recall of 99.70%, F1-score of 99.71%, a false positive rate (FPR) of 0.25%, and a misclassification rate of 0.27%. These findings highlight the crucial role of feature selection in high-dimensional data environments. Future research will investigate how feature selection impacts deep learning architectures, adaptive feature selection, incremental learning approaches, robustness against adversarial attacks, and evaluate model transferability across production web environments to ensure real-time detection reliability, establishing feature selection as a vital step in developing reliable SQL injection detection systems.

## 1 Introduction

In recent years, cyberattacks have grown exponentially, posing significant threats to individuals, organizations, and national infrastructures across the globe (Crespo-Martínez et al., 2023). Their economic toll is estimated at approximately $50 billion annually, with SQL injection attacks making up over 20% of that total (Abad et al., 2023; Bringhenti et al., 2023; Campazas-Vega et al., 2023; Huang et al., 2022). As a result, researchers have explored various methods to detect and prevent SQL injection attacks, including static analysis, rule-based detection approaches, anomaly detection, and supervised machine learning models. These detection techniques, examined by researchers, are generally categorized into three main groups: rule-based approaches (Alenezi et al., 2021; Ciampa et al., 2010), traditional machine learning techniques (Alkhathami and Alzahrani, 2022; Alqahtani et al., 2023; Arasteh et al., 2024), and modern deep learning models (Arasteh et al., 2024; Gowtham and Pramod, 2021; Qu et al., 2024). Despite these advances, a significant theoretical and methodological gap still exists in understanding the role of feature selection as a systematic way to enhance both detection accuracy and computational efficiency.

While rule-based methods can identify certain SQL injection attacks (Bakhsh et al., 2023), they often fail to handle dynamically generated queries and struggle against more sophisticated SQL injection methods (Kumar et al., 2024; Yang et al., 2024). Traditional machine learning techniques, although effective (Souza et al., 2024), face challenges in feature engineering, managing large datasets, and detecting complex or evolving attack patterns (Alkhathami and Alzahrani, 2022; Li et al., 2019; Triloka et al., 2022). Recently, deep learning-based SQL injection techniques have shown significant promise (Alghawazi et al., 2023; Liu and Dai, 2024; Thalji et al., 2023; Paul et al., 2024; Zhang et al., 2022). The performance of these models still lags behind that of state-of-the-art methods. The common limitation across all these categories is the lack of a principled feature selection process to identify the most informative indicators of SQL injection behavior. In effect, the majority of existing studies treat feature extraction as a preprocessing step rather than as a core theoretical component of the detection framework. Few studies have explored feature selection methods for enhancing the performance of machine learning models for SQL injection detection. Among the feature selection approaches investigated, both metaheuristic methods, such as the Gray Wolf Optimizer, and heuristic techniques such as correlation-based selection have been considered (Arasteh et al., 2024; Hassan et al., 2021). However, these studies did not validate the effectiveness of the selected features across various machine learning classifiers, which limits the generalizability and reliability of their findings in diverse classification scenarios. This study positions feature selection not merely as an optimization step but as a central theoretical construct in improving SQL injection detection performance. It argues that feature selection serves as the missing bridge between accuracy and efficiency in this domain. This factor directly determines how well models can generalize to unseen and evolving attack patterns. Therefore, it is crucial to develop more robust solutions for detecting SQL injection attacks in digital security (Arasteh et al., 2024; Qu et al., 2024).

In Tanzania, the majority of websites still rely on rule-based detection approaches, which are often inadequate against modern, dynamic SQL injection threats (Ntembo and Casmir, 2023; Serianu, 2023). Elisa (2020) assessed 79 Tanzania e-government websites and found that over 50% had high-severity vulnerabilities such as SQL injection. Given these persistent challenges and underexplored areas, particularly in feature selection, a novel approach is necessary. To bridge this gap and overcome existing model limitations, this study introduces the DT detection model based on a tree-based architecture. The model begins by extracting features from SQL statements using the Term Frequency-Inverse Document Frequency (TF-IDF) technique, which effectively highlights important information. Next, it applies the Chi-square feature selection method to identify the most significant features for accurate classification.

This study presents an effective SQL injection detection model utilizing a Decision Tree (DT) classifier, which discriminates between malicious and benign SQL statements by recursively partitioning the feature space based on attribute value thresholds. The model leverages DT's capability to handle heterogeneous feature types, capture complex nonlinear decision boundaries, and provide interpretable classification rules with minimal computational overhead during inference. Furthermore, to empirically validate the theoretical premise that feature selection enhances model accuracy, a comparative analysis was conducted between different machine learning models trained with and without Chi-square feature selection, alongside comparisons with findings from existing studies.

## 2 Materials and methods

### 2.1 Datasets

To support this research, a custom dataset (dataset1.csv) was created by capturing normal user payloads from input fields and performing controlled SQL injection attacks on a prototype of the Nelson Mandela African Institution of Science and Technology Research Data Repository (NM-AIST RDR), which was under development during its testing phase. This ensured that the dataset reflected realistic usage scenarios. For malicious queries, SQLMap was run with comprehensive parameters (–tables, –passwords, –current-db, –roles, –columns, –dbs, –schema, –comments, –count, –hostname, –users, –banner, –privileges, –current-user, –is-dba, –dump, –level = 5, –risk = 3, –random-agent, –batch, –answers = “follow = Y”) to generate a wide range of payloads covering all supported techniques (–technique = BEUST for Boolean-based, Error-based, Union-based, Stacked, and Time-based blind injections), providing a full coverage of attack types for model training. Multiple endpoints were targeted using SQLMap's –data parameter, supplying appropriate POST data for each form, to evaluate vulnerabilities across various input fields. The payloads consisted of automated patterns generated by SQLMap, reflecting diverse and realistic attack scenarios. Out-of-band (OOB) exfiltration was configured via a controlled external listener (–dns-domain = <controlled-listener>). The custom dataset was then combined with the SQLiV3.csv dataset (Hussain, 2021) from Kaggle to enhance diversity. The merged dataset initially included 41,573 benign and 40,365 malicious payloads, totaling 81,938 SQL queries before cleaning. After cleaning, the final dataset used for experiments consisted of 34,367 benign and 30,746 malicious queries, totaling 65,113 samples. Within the cleaned dataset, the SQLiV3 subset contributed 19,061 benign (62.6%) and 11,375 malicious (37.4%) queries (30,436 total), while the cleaned custom dataset contributed 15,306 benign (43.6%) and 19,371 malicious (56.4%) queries (34,201 total). These figures show that cleaning and proportional balancing reduced noise and harmonized class representation across sources while maintaining diverse benign and malicious patterns. To evaluate the potential domain shift between the two data sources, a Jensen–Shannon Divergence (JSD) analysis was performed on the TF-IDF feature distributions. The overall JSD between SQLiV3 and the custom dataset was 0.5775 (distance = 0.5775), indicating moderate divergence in feature space. Class-wise analysis yielded a JSD of 0.1340 (distance = 0.3660) for SQL injection (class 1) queries and 0.5320 (distance = 0.7294) for benign (class 0) queries. These results suggest that malicious payloads were relatively consistent across datasets, while benign queries showed moderate lexical variability. This moderate divergence among benign queries is expected in real-world logs, since normal user inputs exhibit high lexical and structural variability across applications, user populations, locales, and input-processing behaviors, which naturally increases distributional heterogeneity relative to crafted malicious payloads. Overall, the combination of real-application captures, SQLMap-generated payloads, and community-sourced samples enhances the dataset's realism and representativeness of practical SQL injection logs; however, it may not fully capture the full diversity of real-world SQL injection activity.

### 2.2 Data preprocessing

Several processes, including data cleaning, label encoding, removing stop words, word segmentation, normalization, building a vocabulary, and TF-IDF feature representation, were performed. The steps were as follows:

- Input: dataset *X* = {*X*_1_, *X*_2_, *X*_3_, …, *X*_*n*_}, label *y* = {*y*_1_, *y*_2_, *y*_3_, …, *y*_*n*_ }.
- Data cleaning: data cleaning was carried out on the SQL injection merged dataset, which involved several activities such as handling missing values, removing duplicates, error rectification, standardizing formatting, and labeling encoding.
- Word segmentation: the text data were divided into individual lexical units or tokens. This step separates continuous text into meaningful words or terms for further analysis.
- Remove stop words: commonly occurring words that carry minimal semantic value, such as “the” and “is,” were removed to reduce noise and enhance the relevance of the remaining text data.
- Normalization: lexical items were normalized by converting them to lowercase, removing special characters, and handling word inflections. This ensures that words with the same meaning but different forms are treated uniformly.
- Build vocabulary: a vocabulary was created by gathering all unique lexical items from all documents. Each token in the vocabulary is assigned a unique index, representing a feature in the model's input matrix.
- Calculate TF-IDF values: the Term Frequency-Inverse Document Frequency (TF-IDF) scores are computed using standard formulas. These values quantify the importance of each word in a document relative to the entire corpus.
- Construct the feature matrix: using the TF-IDF scores, a feature matrix was generated where each row corresponds to a text sample, each column to a vocabulary term, and each element to the TF-IDF value of a word in a document. This matrix (denoted as D) serves as input for subsequent analysis or model training.

The resulting feature matrix D is as follows:

$D=\left[\begin{array}{ccc} a_{11} & \begin{array}{cc} a_{12} & \cdots \end{array} & a_{1k}\\ a_{21} & \begin{array}{cc} a_{22} & \cdots \end{array} & a_{2k}\\ \begin{array}{c} \vdots \\ a_{n1} \end{array} & \begin{array}{c} \begin{array}{cc} \vdots & \;\ddots \end{array}\\ \begin{array}{cc} a_{n2} & \cdots \end{array} \end{array} & \begin{array}{c} \vdots \\ a_{nk} \end{array} \end{array}\right],\;$

where *n* means *n* samples and *k* means *k* vocabulary items (features) are extracted.

- Dataset split: the merged dataset was split into training (80%) and testing (20%) sets using a stratified random split to preserve the proportion of benign and malicious queries across the sets.

### 2.3 Feature selection

Feature selection (FS) is a critical step in reducing irrelevant or excessive features, thereby improving classification accuracy and computational efficiency (Deng et al., 2019; Prastyo et al., 2020). In text classification, features are typically represented as unigrams, n-grams, or parts-of-speech (POS) tags, capturing the essential aspects of documents (Deng et al., 2019). Similarly, in SQL injection detection, features extracted from SQL queries, such as keywords, operators, or token sequences, can be selected using FS techniques to reduce noise and redundancy, which improves the accuracy and efficiency of detection models (Ahmad et al., 2019). FS techniques are generally categorized into filter, wrapper, and embedded methods (Hung et al., 2015). Filter methods, such as Chi-square, Information Gain, and Mutual Information, rely on statistical measures to rank features independently of the classifier (Deng et al., 2019). Wrapper methods evaluate feature subsets based on classifier performance but are computationally expensive and prone to overfitting in high-dimensional, sparse domains such as SQL query logs (Hung et al., 2015; Prastyo et al., 2020). Embedded methods, including LASSO and tree-based models, integrate FS into model training but often require additional hyperparameter tuning and may reduce interpretability (Deng et al., 2019). Prior literature consistently ranks Chi-square alongside Information Gain as one of the most effective filter-based methods for text categorization (Hung et al., 2015). While Information Gain also identifies informative features, Chi-square was preferred since it provides a more robust evaluation for features with skewed distributions, which are common in security-related text data, whereas IG can be biased toward features with higher entropy (Deng et al., 2019). Within this study, the Chi-square test was selected as the FS method due to its efficiency, scalability, and robustness to skewed feature distributions common in SQL query logs. Importantly, preliminary experiments comparing Chi-square with other feature selection (FS) methods, such as Information Gain, Mutual Information, LASSO, and PCA, showed that the Chi-square test produced relatively higher performance metrics for the Multinomial Naïve Bayes classifier, achieving an accuracy of 99.47% and an F1-Score of 99.43%, compared to results with IG (Accuracy 99.40%, F1 99.38%), MI (Accuracy 99.37%, F1 99.37%), LASSO (Accuracy 96.98%, F1 96.98%), and PCA (Accuracy 76.26%, F1 75.51%). These findings provide empirical support for selecting the Chi-square within this study's experimental context. Nonetheless, as the evaluation was limited to Multinomial Naïve Bayes, further validation with additional classifiers is necessary to determine the generalizability of these results across different model architectures. Filter-based methods preserve the original feature interpretability, which is essential in security analysis, where understanding which query terms contribute to classification is as important as achieving high accuracy (Deng et al., 2019). To enhance the effectiveness of SQL injection detection by eliminating feature redundancy, this study used the Chi-square test with Multinomial Naïve Bayes as a baseline model, creating a reliable way to select the top k features. Multinomial Naïve Bayes was chosen for its simplicity, speed, low resource requirement, and effectiveness with higher-dimensional data.

### 2.4 Chi-square feature selection

The Chi-square test is a statistical method used to determine whether there is a significant association between two categorical variables. It is often used to evaluate the relevance of a feature to the target class. The test usually compares the observed frequency of an event (how often it happens) with the expected frequency (how often would it happen if there were no relationship). If the observed and expected values are very different, the feature is likely important. It is given by *X*^2^ in Equation 1.


$$\begin{array}{l} X^{2}=\;\overset{n}{\underset{i=1}{\sum }}\overset{m}{\underset{j=1}{\sum }}\frac{{\left(O_{ij}-E_{ij}\right)}^{2}}{E_{ij}}, \end{array}$$ (1)

where *O*_*ij*_ is the observed frequency of feature *i* in class *j, E*_*ij*_ is the expected frequency of feature *i* in class *j* (assuming independence), *n* is the number of feature categories, and *m* is the target class. Furthermore, *E*_*ij*_is given as in Equation 2.


$$\begin{array}{l} E_{ij}=\frac{\left({RowTotal}_{i}\right)\times \left({Column\;Total}_{j}\right)}{GrandTotal} \end{array}$$ (2)

### 2.5 Identification of top-k features

To identify the most informative features for SQL injection detection, a two-step top-*k* feature selection process was used, combining the Chi-square test with the Multinomial Naïve Bayes classifier. First, the dataset was divided into 80% training and 20% testing sets using stratified random sampling, and textual features were represented with TF-IDF vectors.

Coarse search: a broad search evaluated model performance at intervals of 50 features, from the first 50 up to the total number of features. For each candidate *k*, the top *k* features were selected using the Chi-square test, and a Multinomial Naïve Bayes model was trained and tested. The feature count that yielded the highest accuracy was recorded as the preliminary best *k*.

Fine search: a detailed search was then performed around the preliminary best *k* (±200 features) with a step size of 1 to more precisely examine performance trends. Instead of focusing on a single value of *k*, this step revealed a plateau region, where multiple consecutive *k* values achieved the same maximum accuracy. This plateau indicates that the model's performance is stable across that range, allowing flexibility in choosing *k* without sacrificing accuracy. Highlighting this plateau ensured that the final feature set was not restricted to a single arbitrary cutoff but rather selected from a range of equally optimal feature counts. This balances informativeness and size, enhancing classification accuracy while keeping computational demands manageable. The process was visualized by plotting accuracy against the number of selected features for both the coarse and fine searches, with the plateau region shaded to clearly show the stability zone for top-*k* features.

### 2.6 Data visualization

This study employed a visualization technique to understand the distribution of data points across the dataset before and after feature selection. The t-SNE algorithm was used to visualize the feature vectors in two dimensions from high-dimensional data. T-SNE is known for its ability to reveal significant global relationships, while preserving the local structure of the data.

### 2.7 Machine learning model

This study developed five machine learning models for SQL injection detection, with and without Chi-square feature selection. These models included Multinomial Naïve Bayes, Decision Tree, Logistic Regression, Support Vector Machine, and K-Nearest Neighbor. To enhance the robustness of model evaluation, stratified k-fold cross-validation (*k* = 5) was applied instead of relying solely on a single train-test split. Stratification preserved the original query-type proportions across all folds, ensuring that both training and validation subsets maintained identical class balance. The full dataset contained 30,746 SQL injection (malicious) queries (47.24%) and 34,367 benign queries (52.76%), totaling 65,113 samples. Folds 1–4 each contained a training set with 24,597 SQL injection queries (47.24%) and 27,494 benign queries (52.76%) and a testing set with 6,149 SQL injection queries (47.24%) and 6,873 benign queries (52.76%). Fold 5 contained a training set with 24,598 SQL injection queries (47.24%) and 27,493 benign queries (52.76%) and a testing set with 6,148 SQL injection queries (47.23%) and 6,874 benign queries (52.77%). These consistent class proportions across all folds confirm that stratification effectively preserved the original dataset distribution, preventing bias and ensuring fair model evaluation. For each iteration, four folds were used for training, while the remaining fold was used for validation. This process was repeated five times, each with a different validation fold, and the results were aggregated and reported as mean ± standard deviation (SD) to capture both central tendency and variability across folds. By averaging performance across multiple folds, cross-validation reduces the risk of overfitting to a particular split and provides a more reliable estimate of generalization performance. Additionally, further analysis was conducted on the best-performing model to gain more insights, which involved error analysis and testing the model on external data. The sql injection dataset (sqli.csv) from the Kaggle repository was used as external data. It originally contained 4,200 entries (1,128 SQL injection queries and 3,072 normal queries). After data cleaning, including the removal of duplicates and ambiguous entries, 3,951 unique entries remained and were used for analysis.

#### 2.7.1 Multinomial Naïve Bayes (MNB)

MNB is a probabilistic classifier based on Bayes' theorem that assumes conditional independence between features. It is effective for text classification tasks involving discrete data, such as term frequencies in documents. The probability of a class *c* given the document *d* is calculated as in Equation 3.


$$P\left(c|d\right)\propto P\left(c\right)\prod_{i=1}^{n}P(w_{i}|c{)}^{f_{i}}\;,$$ (3)

where *P(c)* indicates prior probability of class *c, w*_*i*_ indicates word *i, fi* indicates frequency of word *wi* in document *d*, and *P (wi| c)* is the probability of word *wi* occurring in class *c*.

#### 2.7.2 Logistic regression (LR)

This is a linear classification model that predicts the class membership using the logistic (sigmoid) function. It is commonly applied to binary and multiclass classification problems due to its efficiency and interpretability. It is given by the formula *P(y* = *1|x)* in Equation 4:


$$\begin{array}{l} P\left(y=1|\;x\right)=\sigma \left(w^{T}+b\right)=\;\frac{1}{1+e^{-\left(w^{T}\;x+b\right)}}\;, \end{array}$$ (4)

where *w* is the weight vector, *x* is the input feature vector, *b* is the bias term, and σ is a sigmoid function.

#### 2.7.3 Decision tree (DT)

This is the tree-based classifier that splits the dataset into branches to form a hierarchy of decision rules. It utilizes a tree-like model of decisions based on feature values. The trees are created using the information gain calculation Equations 5, 6.


$$\begin{array}{l} Entropy\;\left(S\right)=\;-\overset{c}{\underset{i=1}{\sum }}p_{i}\log_{2}\left(pi\right), \end{array}$$ (5)

$$\begin{array}{l} \;Information\;Gain\;\left(S,A\right)\\ =Entropy\left(S\right)-\;\sum_{u\in Values\;\left(A\right)}\frac{\left|S_{V}\right|}{S}Entropy\;(S_{V}), \end{array}$$ (6)

where *S* is the dataset, *A* is an attribute, and *S*_*V*_ is a subset of S where attribute *A* = *v*.

#### 2.7.4 Support vector machine (SVM)

This is a supervised machine learning classifier that seeks to identify the optimal hyperplane that maximizes the margin between class boundaries. It works well for both linear and non-linear classification tasks, especially in high-dimensional feature spaces. The study employed linear SVM since the nature of the dataset was binary. The decision function is used to reflect how far the input data point is from the hyperplane, which is the optimal operator between the two classes. The decision function is given by Equation 7:


$$\begin{array}{l} f\left(x\right)=sign\;\left(w^{T}x+b\right), \end{array}$$ (7)

and the optimization problem is


$$\frac{1}{2}||w|{|}^{2}subject\;to\;y_{i}(w^{T}x_{i}+b)\geq \forall_{i},$$ (8)

where *w* is the weight vector (normal to the hyperplane), *x* is the feature vector of the input data point, *b* is the bias term (intercept), and *y*∈{−1, 1} is a true class label.

#### 2.7.5 K-Nearest Neighbors (KNN)

This is a non-parametric, instance-based learning algorithm that classifies data points based on the majority class among their k nearest neighbors in feature space. It is sometimes called a lazy learner because it does not learn during training. This classifier relies on distance measurements, usually using the Euclidean distance formula, as shown in Equation 9.


$$d\left(x,x_{i}\right)=\sqrt{\sum_{j=1}^{n}{\left(x_{j}-x_{ij}\right)}^{2}},$$ (9)

where *x* is the query point, *x*_*i*_ is the *i*th training instance, and *x*_*j*_ is the *j*th feature of *x*. The predicted class is the most frequent class among the k nearest neighbors.

### 2.8 Model evaluation metrics

To evaluate the performance of each classifier, various evaluation metrics such as accuracy, precision, recall, F1-score, false positive rate, and misclassification rates were employed during the evaluation phase. These metrics provided a base for comparisons of these machine learning classifiers using a set of four distinct combinations, represented by the symbols True Positive (TP), False Positive (FP), True Negative (TN), and False Negative (FN). In this context, true means the values were accurately classified, and false means the values were incorrectly classified.

The accuracy of the classifier measures how well the model classifies the input query as SQL injection or normal. It can be determined using the formula shown in Equation 10.


$$\begin{array}{l} Accuracy=\frac{TP+TN}{TP+TN+FP+FN} \end{array}$$ (10)

Precision is used to measure the number of classified positive cases that were positive. High precision means fewer false positives. It is given by the formula shown in Equation 11.


$$\begin{array}{l} Precision=\frac{TP}{TP+FP} \end{array}$$ (11)

Recall is the evaluation metric that measures the sensitivity of positive cases, that is, how many positive cases were correctly classified. It is given by the formula shown in Equation 12.


$$\begin{array}{l} Recall=\frac{TP}{TP+FN} \end{array}$$ (12)

The F1-score is the evaluation metric that considers both precision and recall to give a balanced assessment of the model's performance. It penalizes the extreme value more than the arithmetic mean to ensure that both precision and recall contribute equally. It is given by the formula shown in Equation 13.


$$\begin{array}{l} F1-Score=\frac{2^{*}recall^{*}precision}{recall+precision} \end{array}$$ (13)

False Positive Rate (FPR) is the proportion of actual negative instances that are incorrectly labeled as positive by the classifier. It is given by the formula in Equation 14.


$$\begin{array}{l} FPR=\frac{FP}{TN+FP} \end{array}$$ (14)

Misclassification measures how the model was unable to classify the query correctly. It is given by the formula in Equation 15.


$$\begin{array}{l} Misclassification\;rate=\frac{FP+FN}{TP+TN+FP+FN} \end{array}$$ (15)

## 3 Results

### 3.1 Optimal feature set selection results

**Figure 1:**
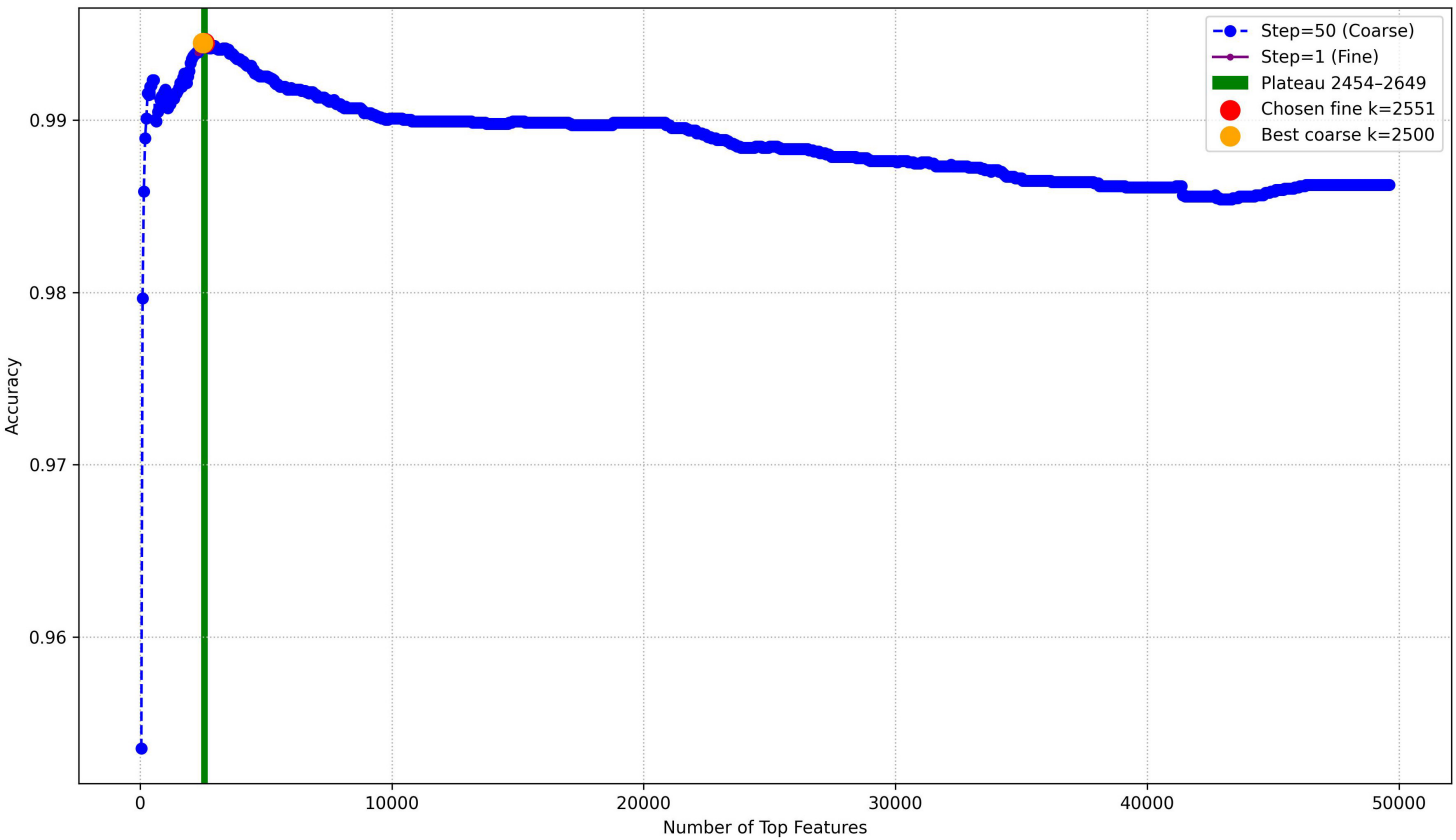
Model accuracy vs. number of Chi-square selected top-k features, showing the plateau region used to identify the optimal feature range.

**Figure 2:**
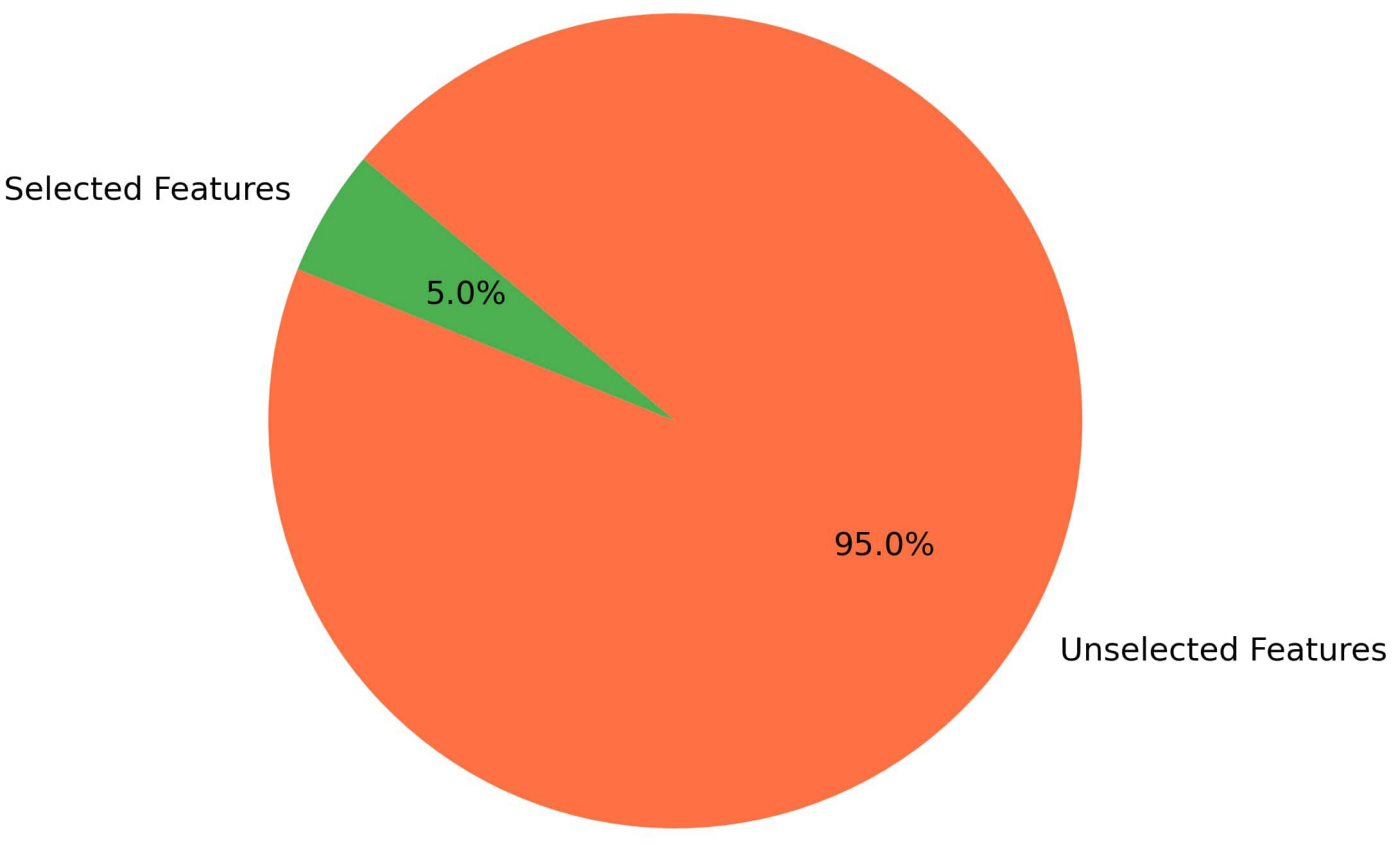
Percentage of selected features.

The results showed that model performance steadily improved as the number of features increased, reaching a peak at *k* = 2,454. Beyond this point, accuracy plateaued up to *k* = 2,649, after which the performance slightly declined due to the inclusion of noisy or redundant features. Choosing the midpoint of the plateau (*k* = 2,551, as identified in the fine search) offered the best balance between accuracy and model efficiency, preventing unnecessary feature overhead while maintaining optimal performance. This demonstrates that Chi-square feature selection effectively reduced the dimensionality of SQL query logs while maintaining the most discriminative terms for classification. As shown in Figure 1, the accuracy curve increases sharply before leveling off, with the best point marked at *k* = 2,551, compared to the coarse peak at *k* = 2,500, beyond which performance improvements diminished. Based on this curve, the top 2,551 features, approximately 5% of the total 49,607 features, were selected and retained, while the remaining features were not, as shown in the pie chart in Figure 2.

### 3.2 T-SNE visualization results

**Figure 3:**
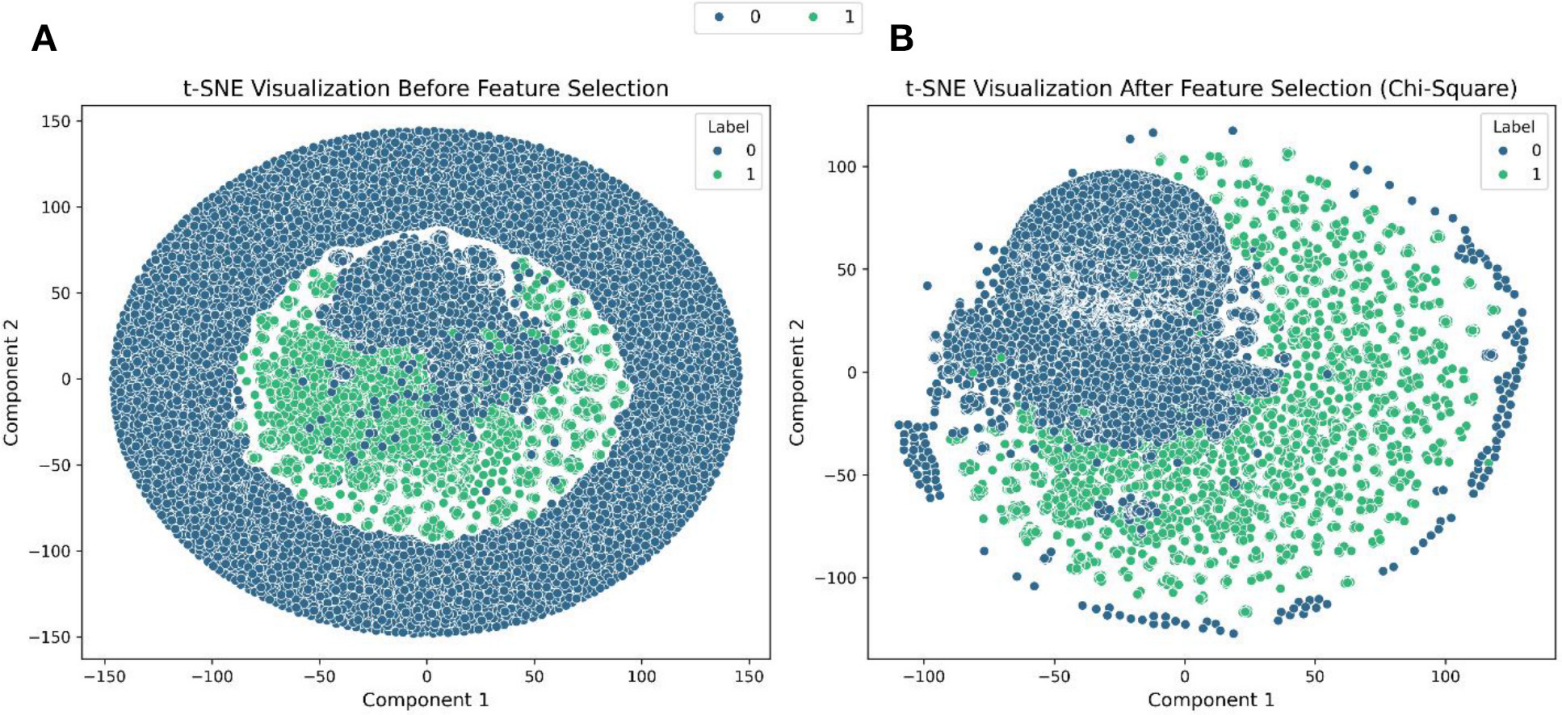
T-SNE visualization results. **(A)** Before feature selection. **(B)** After feature selection.

Figure 3 shows the effects of dimensionality reduction before and after feature selection. Before feature selection, the dataset exhibited significant overlap between the two classes, as observed in Figure 3A. Data points corresponding to Label 0 (Normal) and Label 1 (SQLi) were highly intermixed, particularly in the central region of the plot, indicating poor class separability. This suggests that the original feature space contained irrelevant or redundant information that hindered the model's ability to distinguish between the classes effectively. However, after applying the Chi-Square test, a marked improvement in class separability was observed, as shown in Figure 3B. The t-SNE visualization of the reduced feature space revealed more distinct clusters, with data points of each class forming clearer and more compact groupings. This enhanced separation indicates that the selected features preserved the most informative aspects of the data, reducing noise and improving the dataset's structure. Consequently, the feature selection process not only simplified the model but also contributed to better discrimination between classes, which is expected to enhance the overall performance of the classification model.

### 3.3 Comparison of models' classification results

**Figure 4:**
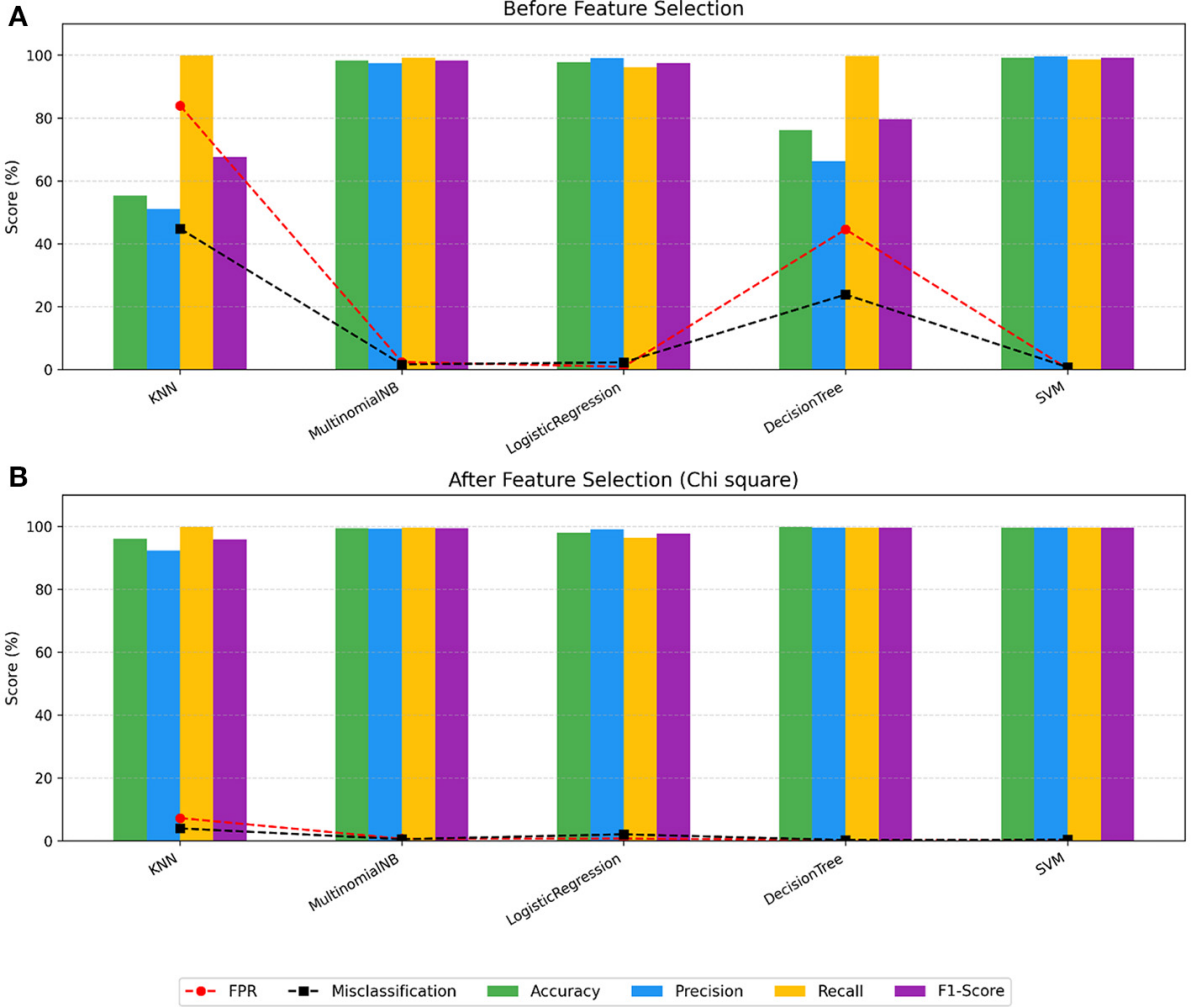
Classifiers' performance dynamics. **(A)** Before feature selection. **(B)** After feature selection (Chi-square).

Before feature selection (Table 1 and Figure 4A), SVM, LR, and MNB performed well across all metrics. Meanwhile, the SVM model achieved the highest accuracy (99.23%), precision (99.65%), recall (98.70%), and F1-Score (99.17%), along with the lowest FPR (0.30%) and misclassification rate (0.77%). In contrast, DT and KNN showed the poorest performance on most metrics, with KNN recording the lowest accuracy (55.22%), precision (51.08%), and F1-score (61.57%), as well as the highest FPR (83.93%) and misclassification rate (44.78%).

**Table 1:** Models' performance before feature selection.

**Model**	**Accuracy (%)**	**Precision (%)**	**Recall (%)**	**F1-Score (%)**	**FPR (%)**	**Misclassification (%)**
MNB	98.36	97.34	99.21	98.27	2.38	1.64
LR	97.73	98.97	96.14	97.53	0.88	2.27
DT	76.17	66.28	99.77	79.65	44.55	23.83
SVM	99.23	99.65	98.70	99.17	0.30	0.77
KNN	55.22	51.08	99.82	67.57	83.93	44.78

After applying the Chi-square feature selection (Table 2 and Figure 4B), all models demonstrated significant improvements. The DT classifier advanced from second-worst to the top performer, achieving an accuracy of 99.73%, a precision of 99.72%, a recall of 99.70%, an F1-score of 99.71%, a FPR of 0.25%, and a misclassification rate of 0.27%. Similarly, KNN improved substantially, though the lowest performer, with an accuracy of 96.04%, a precision of 92.35%, a recall of 99.79%, an F1-score of 95.93%, a FPR of 7.25%, and a misclassification rate of 3.96%.

**Table 2:** Models' performance after feature selection (Chi-square).

**Model**	**Accuracy (%)**	**Precision (%)**	**Recall (%)**	**F1-Score (%)**	**FPR (%)**	**Misclassification (%)**
MNB	99.47	99.25	99.62	99.43	0.66	0.53
LR	97.92	99.12	96.40	97.74	0.75	2.08
DT	99.73	99.72	99.70	99.71	0.25	0.27
SVM	99.59	99.59	99.54	99.56	0.36	0.41
KNN	96.04	92.35	99.79	95.93	7.25	3.96

**Figure 5:**
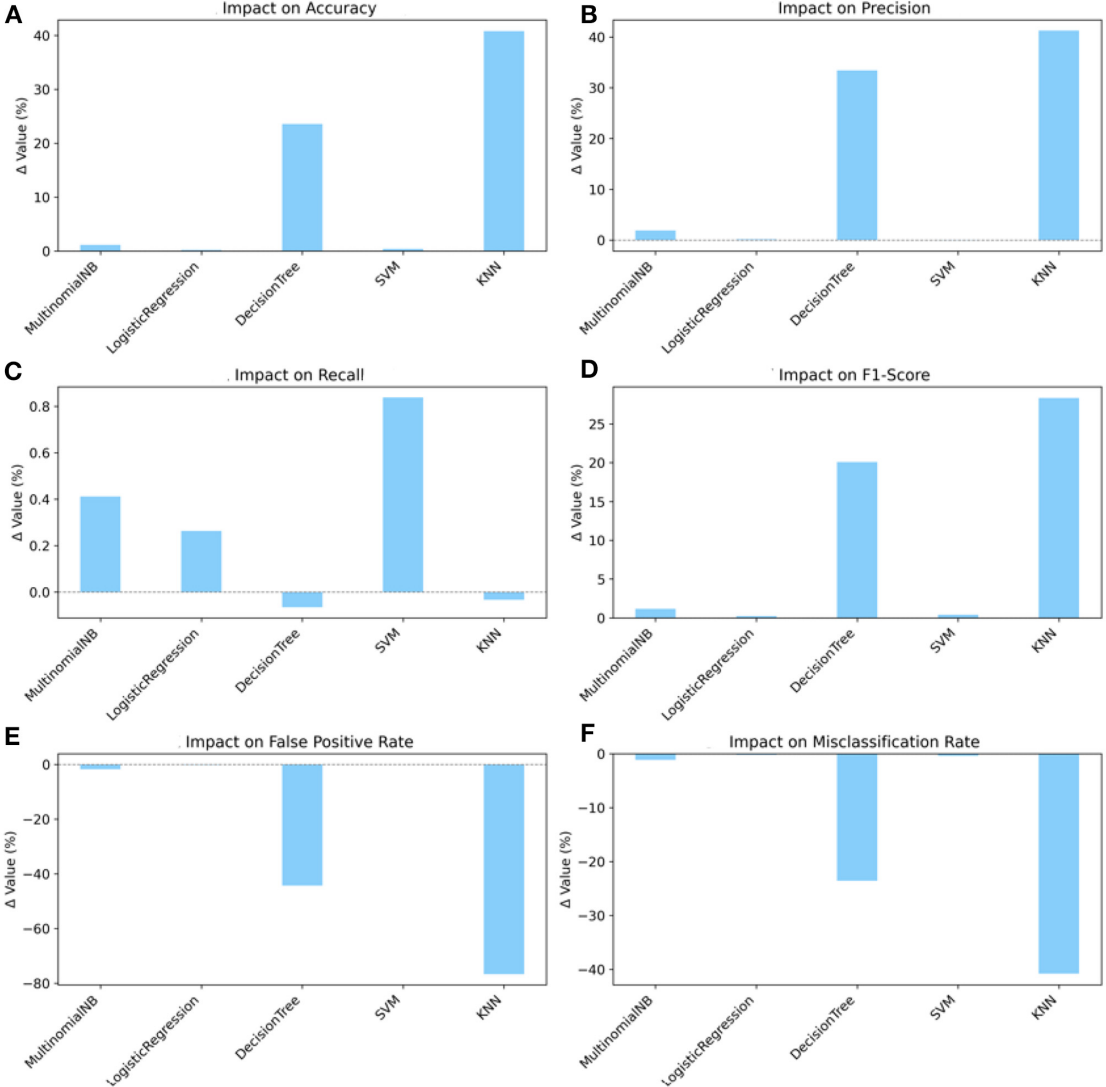
Impact of chi-square feature selection on classifiers' performance. **(A)** Impact on accuracy. **(B)** Impact on precision. **(C)** Impact on recall. **(D)** Impact on F1-score. **(E)** Impact on false positive rate. **(F)** Impact on misclassification rate.

Chi-square positively impacted all models, with KNN benefiting the most. Its accuracy increased by 40.82%, precision by 41.27%, and F1-score by 28.36%, along with a significant reduction in FPR and misclassification rate of 76.68% and 40.82%, respectively. Meanwhile, LR showed slight but consistent improvements: increases in accuracy of 0.19%, precision of 0.15%, recall of 0.26%, and F1-score of 0.21%, respectively, and reductions in FPR and misclassification rate of 0.13% and 0.19%, respectively, as shown in Figures 5A–F.

Feature selection significantly improved the computational efficiency of all models, as shown in Table 3. Training time was highest for SVM (837.49 s before, 71.56 s after) and lowest for KNN (0.0087 s before, 0.0076 s after). Inference time per query was highest for KNN (1.966 ms before, 1.891 ms after) and lowest for LR (0.000106 ms before, 0.000089 ms after). Peak memory usage was highest for SVM (23.70 MB before, 20.36 MB after) and lowest for MNB (3.51 MB before, 2.88 MB after). Model size was highest for KNN (6.27 MB before and after) and lowest for MNB (1.31 MB before, 0.08 MB after). Overall, feature selection reduced training time, memory footprint, and model size, while maintaining efficient inference across all models.

**Table 3:** Runtime and memory usage of models before and after feature selection.

**Model**	**Training time (s)**		**Inference time (ms/query)**		**Peak memory (MB)**		**Model size (MB)**	
	**Before**	**After**	**Before**	**After**	**Before**	**After**	**Before**	**After**
MNB	0.018299	0.014423	0.000248	0.000161	3.51	2.88	1.31	0.08
LR	0.991392	0.206367	0.000106	0.000089	14.39	2.40	0.33	0.02
DT	61.112381	3.991875	0.017937	0.00963	9.84	8.09	1.95	0.10
SVM	837.489763	71.556955	0.528554	0.126762	23.70	20.36	1.04	0.26
KNN	0.008689	0.007574	1.965834	1.890653	6.28	5.44	6.27	4.34

### 3.4 Cross-validation results for selected features

The results from five-fold cross-validation, following Chi-square feature selection, aligned closely with those from the single train-test split, showing only slight variations across different folds (Table 4). The cross-validation outcomes confirmed that the model's performance was consistent across folds, indicating that the results did not depend on a specific train-test split. Decision Tree (DT) achieved the highest performance with an accuracy of 99.70 ± 0.04%, a precision of 99.66 ± 0.05%, a recall of 99.71 ± 0.10%, an F1-score of 99.68 ± 0.05%, a false positive rate of 0.31 ± 0.05%, and a misclassification rate of 0.30 ± 0.04%. Multinomial Naïve Bayes (MNB) and Support Vector Machine (SVM) also performed very well, with an accuracy above 99%, precision and recall above 99%, and low error rates. Logistic Regression (LR) remained steady at an accuracy of 98.04 ± 0.12%, with a precision of 99.09 ± 0.07%, a recall of 96.73 ± 0.28%, an F1-score of 97.90 ± 0.13%, a false positive rate of 0.79 ± 0.06%, and a misclassification of 1.96 ± 0.12%. K-Nearest Neighbors (KNN), though significantly improved after feature selection, was still the lowest-performing model, with an accuracy of 95.63 ± 0.28%, a precision of 91.73 ± 0.52%, a recall of 99.75 ± 0.08%, an F1-score of 95.57 ± 0.27%, a false positive rate of 8.05 ± 0.55%, and a misclassification rate of 4.37 ± 0.28%. Overall, these results demonstrate that the feature selection method is robust, and the model's performance is consistent across various data folds.

**Table 4:** Models' cross-validation results after feature selection.

**Model**	**Accuracy (%)**	**Precision (%)**	**Recall (%)**	**F1-score (%)**	**FPR (%)**	**Misclassification (%)**
MNB	99.46 ± 0.05	99.24 ± 0.09	99.63 ± 0.04	99.43 ± 0.05	0.68 ± 0.08	0.54 ± 0.05
LR	98.04 ± 0.12	99.09 ± 0.07	96.73 ± 0.28	97.90 ± 0.13	0.79 ± 0.06	1.96 ± 0.12
DT	99.70 ± 0.04	99.66 ± 0.05	99.71 ± 0.10	99.68 ± 0.05	0.31 ± 0.05	0.30 ± 0.04
SVM	99.62 ± 0.06	99.64 ± 0.11	99.54 ± 0.05	99.59 ± 0.06	0.32 ± 0.10	0.38 ± 0.06
KNN	95.63 ± 0.28	91.73 ± 0.52	99.75 ± 0.08	95.57 ± 0.27	8.05 ± 0.55	4.37 ± 0.28

### 3.5 Comparative results with other related works

The proposed model's performance was evaluated against various related studies, as shown in Table 5. While deep learning approaches may achieve comparable or superior generalization on larger, heterogeneous datasets, our results show that feature selection with classical ML can achieve competitive accuracy (99.73%) and F1-score (99.71%) with lower computational costs (training time 3.99 s, inference time 0.00963 ms/query, peak memory 8.09 mb, and model size of 0.1 mb). However, these results should be interpreted considering variations in datasets, preprocessing steps, and algorithmic architectures across studies that may have influenced the comparative outcomes. For example, Chen et al. (2021) and Falor et al. (2021) utilized deep learning architectures such as convolutional neural networks (CNNs) trained on tens of thousands of labeled SQL injection and benign samples, focusing on hierarchical feature learning from sequential data. Similarly, Potinteu and Varga (2020), Joshi et al. (2022), and Takyi et al. (2025) employed LSTM-based models capable of capturing temporal dependencies. These methods achieved high accuracy (91%−99.4%) but required large datasets and significant computational resources during training and deployment. Conversely, researchers such as Shakya et al. (2024), Jothi et al. (2021), and Crespo-Martínez et al. (2023) used classical machine learning algorithms such as KNN, SVM, Logistic Regression, and Random Forests, which are more computationally efficient and easier to interpret but often less effective at modeling complex features. The improved performance of the proposed model is credited to the use of feature selection techniques, which enhanced learning efficiency by removing redundant or irrelevant attributes, resulting in more stable classification boundaries and better generalization without the high computational costs associated with deep learning. From a deployment perspective, this approach is lightweight and suitable for real-time or resource-limited environments such as institutional web applications and local information systems. In conclusion, while the proposed model performed well, its advantages should be considered within the context of dataset variability, preprocessing methods, and algorithmic architectures.

**Table 5:** Comparative results with other related works.

**Authors**	**Accuracy**	**Precision**	**Recall**	**F1-score**
Potinteu and Varga (2020)	96%	90%	74%	81%
Jothi et al. (2021)	98%	98%	97%	–
Chen et al. (2021)	98.57%	97.95%	99.22%	98.58%
Falor et al. (2021)	94%	85%	96%	–
Joshi et al. (2022)	91%	91%	91%	91%
Crespo-Martínez et al. (2023)	97.23%	–	97.3%	97.3%
Shakya et al. (2024)	95.55%	99%	–	–
Takyi et al. (2025)	99.4%	99.4%	99.4%	99.4%
Proposed model	99.73%	99.72%	99.70%	99.71%

### 3.6 Additional analysis of the proposed model

#### 3.6.1 Error analysis results

The error analysis conducted on the proposed model revealed only a small number of misclassifications, with counts distributed evenly across SQLi types. Tautology-based, boolean-based, time-based blind, and obfuscated queries each produced two errors, while union-based, stacked queries, comment-based, error-based, and function-based attacks contributed one each. This balanced pattern indicates strong generalizability, as no single attack type emerged as a systematic weakness. To further illustrate, a tautology-based query such as (‘)) AS JPcN WHERE 9939 = 9939 OR NOT 5463 = 5463) was occasionally misclassified as benign due to its high lexical overlap with non-malicious inputs containing conditional expressions. Similarly, the boolean-based payload (select case when 6420 = 7941 then 1 else null ...) was misclassified because its conditional syntax closely resembles legitimate SQL logic. An obfuscated fragment ('JMoRSq <'“>SPXBve) also led to errors, as its irregular token structure hindered proper feature extraction. A union-based example, union select 1, load_file('/etc/passwd'), was misclassified in one instance, likely because the presence of file-reading function calls and the union operator created token patterns that partially overlapped with complex but benign multi-select statements. These examples highlight the model's limitations in distinguishing between structurally valid yet semantically malicious queries and in handling heavily distorted lexical patterns not fully captured by the TF–IDF and Chi-Square feature selection methods.

#### 3.6.2 External data testing results

The external data testing results showed that the model maintained a high performance, achieving an accuracy of 99.76%, a precision of 99.66%, a recall of 99.84%, and an F1-score of 99.75%, with a false positive rate of 0.30% and a misclassification rate of 0.24%. These results demonstrate the model's ability to preserve its detection strength on unseen data. However, it should be noted that the external dataset used for testing (sqli.csv) and a portion of the data used for model training (SQLiV3) were both obtained from the Kaggle repository on sql injection dataset. While the datasets are independent, they may share similar characteristics or patterns due to being collected from related environments. Therefore, further validation on more diverse and heterogeneous datasets is recommended to fully assess the model's generalization capability.

## 4 Discussion

The experimental results showed that Chi-square feature selection improved model performance across all classifiers. Notably, DT and KNN demonstrated substantial improvement from poor to excellent performance. MNB, LR, and SVM also improved to a smaller extent, indicating that feature selection reduced noise and redundancy across the dataset. The impact of Chi-square feature selection varied across classifiers, which can be due to differences in how algorithms handle high-dimensional and noisy data. Models such as Decision Tree (DT) and K-Nearest Neighbors (KNN) are very sensitive to irrelevant or redundant features. For DT, too many features often lead to fragmented splits and overfitting, as the model greedily splits the data even when the selected attributes lack discriminative power. Reducing the feature space makes sure splits are based only on statistically significant attributes, which stabilizes the tree structure and enhances generalization. Similarly, the substantial improvement observed in the K-Nearest Neighbors (KNN) classifier after applying Chi-Square feature selection can be explained by the algorithm's sensitivity to irrelevant and noisy features. Before feature selection, KNN computed distances across thousands of TF-IDF features, including many non-informative SQL tokens such as FROM, SELECT, WHERE, AND, and NULL, as well as punctuation-based tokens like /^*^…^*^/. These common structural terms occurred frequently in both benign and malicious queries, artificially inflating the feature space and distorting Euclidean distance calculations. For instance, the query SELECT pg_sleep(5) AND (((“wqeb” = “wqeb”), a typical time-based SQL injection, was misclassified as benign because irrelevant structural and string tokens masked the discriminative feature. After applying Chi-square selection, the retained query became select pg_sleep and, which preserved the key token pg_sleep, allowing the model to identify the query as malicious correctly. Similarly, the conditional query AND 6692 = (SELECT (CASE WHEN (6692 = 2402) THEN 6692 ELSE (SELECT 2402 UNION SELECT 3794) END)) was correctly reclassified after irrelevant numeric tokens were removed. This reduction in feature noise effectively minimized the curse of dimensionality, improved distance-based neighborhood formation, and enhanced KNN's ability to group similar samples accurately. In contrast, models such as Support Vector Machine (SVM) and Logistic Regression (LR) are more immune to high-dimensional noise. SVM primarily depends on support vectors and finds the optimal decision boundary by maximizing the margin, which lessens the impact of irrelevant features. Logistic Regression can shrink unhelpful coefficients, reducing the effects of noisy dimensions. As a result, these models benefit from feature selection but only see slight improvements in classification performance. Multinomial Naïve Bayes (MNB) falls between these groups. Although generally resistant to high-dimensional input because of its probabilistic approach, MNB still gains from feature selection because removing redundant terms sharpens class-conditional probability distributions.

The near-perfect performance observed, particularly for the Decision Tree model (99.73% accuracy), indicates that the models effectively captured discriminative patterns in the SQL query logs. Although such high accuracy could suggest potential overfitting given the high-dimensional TF-IDF features that may encourage memorization, the consistency observed across five-fold cross-validation, together with the small and evenly distributed misclassification counts for SQL injection queries in the Decision Tree error analysis, mitigates this concern. Evaluation on an external dataset produced comparable results, further supporting the generalizability of the models. Chi-square feature selection contributed by filtering out irrelevant and redundant features, allowing the models to focus on the most informative terms. However, in realistic deployment scenarios, factors such as unseen query structures, evolving attack patterns, and noisy web traffic could slightly lower performance. These results align with existing studies (Gnana et al., 2016; Ahmad et al., 2019; Deng et al., 2019; Qin et al., 2025), which highlight that feature selection enhances performance when working with high-dimensional data, such as the SQL injection detection task and models prone to overfitting due to irrelevant features. The significant improvement in DT and KNN reinforces earlier findings that such models are more sensitive to high-dimensional input, and the marginal yet consistent gain in MNB, SVM, and LR supports their known robustness, even in the presence of noisy features. While feature extraction methods, such as Bag-of-Words and TF-IDF, are essential for transforming raw text into a numerical form, they often produce high-dimensional representations. Feature selection, in contrast, plays a critical role in identifying the most relevant features from these representations, thereby improving model efficiency and generalizability. The study contributes to the existing body of knowledge by offering empirical evidence that Chi-square feature selection not only enhances underperforming models but also refines the output of high-performing models.

From a deployment perspective, feature selection also contributes to practical efficiency in runtime and memory usage. In real-world web applications where SQL queries must be inspected in real time, reducing the number of processed features directly translates to lower inference latency and faster decision-making. This improvement is critical for online detection systems, which must handle high query volumes without degrading user experience or backend performance. Moreover, compact feature subsets make models easier to update incrementally as new SQL variants emerge, which supports adaptive detection pipelines. Another key implication is security robustness. Attackers may craft obfuscated or polymorphic SQL injection payloads that differ syntactically but retain malicious intent. The findings suggest that feature selection can strengthen the model's focus on semantic indicators rather than the superficial syntax, potentially increasing resilience against such variants. This underlines the importance of integrating optimized feature subsets into deployment frameworks that must balance detection accuracy, computational efficiency, and resistance to adversarial inputs.

## 5 Conclusion and future work

This study addresses the research problem of evaluating the impact of feature selection on detecting SQL injection attacks in web applications. The primary goal of the study is to demonstrate how accurate feature selection can improve the performance of machine learning models in identifying SQL injections. Key findings indicate that feature selection has a significantly positive effect, substantially enhancing all models by removing noisy and irrelevant features. These findings imply that redundant and irrelevant features generate noise and reduce generalizability; therefore, feature selection should be prioritized before model training, particularly for high-dimensional datasets. This study highlights that the limited exploration of feature selection in prior works represents a critical theoretical gap in the current body of knowledge. By systematically demonstrating that optimized feature subsets can yield higher accuracy and lower false positive rates across models, this research provides empirical evidence that the discriminative power of features, not just model complexity, determines detection success. Consequently, feature selection emerges as the missing bridge between accuracy, interpretability, and computational efficiency in machine learning-based SQL injection detection. This research has limitations, including the need to validate the selected feature selection method on different SQL injection multiclass data and other non-SQL injection attack types. Further testing on independent real-world data is required to confirm robustness. Although query types were balanced and datasets randomized, residual differences between the custom dataset and SQLiV3 may still affect generalizability. Additionally, real-time deployment requires low-latency detection (typically <200 ms per query) and resilience to evolving obfuscation patterns. As the inference time for the proposed model was 0.00963 ms/query, practical deployment challenges, such as real-time detection latency, evolving query obfuscation, and adaptive adversaries, were not explored in this study. Future research should address these considerations, including integrating feature selection with deep learning architectures, developing adaptive and incremental learning approaches, improving robustness against adversarial SQL injection variants, and evaluating model transferability across production web environments to ensure real-time detection reliability. Overall, feature selection is essential for boosting classification accuracy and reducing false positives in SQL injection detection, particularly with sparse data.

## Data Availability

The raw data supporting the conclusions of this article will be made available by the authors, without undue reservation.
